# Stimulation of Surface Polysaccharide Production under Aerobic Conditions Confers Aerotolerance in Campylobacter jejuni

**DOI:** 10.1128/spectrum.03761-22

**Published:** 2023-02-14

**Authors:** Jinshil Kim, Myungseo Park, Eunbyeol Ahn, Qingqing Mao, Chi Chen, Sangryeol Ryu, Byeonghwa Jeon

**Affiliations:** a Department of Food and Animal Biotechnology, Research Institute of Agriculture and Life Sciences, Seoul National University, Seoul, Republic of Korea; b Department of Agricultural Biotechnology, Seoul National University, Seoul, Republic of Korea; c Center for Food and Bioconvergence, Seoul National University, Seoul, Republic of Korea; d Division of Environmental Health Sciences, School of Public Health, University of Minnesota, Minneapolis, Minnesota, USA; e Department of Food Science and Nutrition, University of Minnesota, Saint Paul, Minnesota, USA; South China Sea Institute of Oceanology

**Keywords:** *Campylobacter*, oxygen sensitivity, aerotolerance, surface polysaccharides

## Abstract

The ability of a foodborne pathogen to tolerate environmental stress critically affects food safety by increasing the risk of pathogen survival and transmission in the food supply chain. Campylobacter jejuni, a leading bacterial cause of foodborne illnesses, is an obligate microaerophile and is sensitive to atmospheric levels of oxygen. Currently, the molecular mechanisms of how C. jejuni withstands oxygen toxicity under aerobic conditions have not yet been fully elucidated. Here, we show that when exposed to aerobic conditions, C. jejuni develops a thick layer of bacterial capsules, which in turn protect C. jejuni under aerobic conditions. The presence of both capsular polysaccharides and lipooligosaccharides is required to protect C. jejuni from excess oxygen in oxygen-rich environments by alleviating oxidative stress. Under aerobic conditions, C. jejuni undergoes substantial transcriptomic changes, particularly in the genes of carbon metabolisms involved in amino acid uptake, the tricarboxylic acid (TCA) cycle, and the Embden-Meyerhof-Parnas (EMP) pathway despite the inability of C. jejuni to grow aerobically. Moreover, the stimulation of carbon metabolism by aerobiosis increases the level of glucose-6-phosphate, the EMP pathway intermediate required for the synthesis of surface polysaccharides. The disruption of the TCA cycle eliminates aerobiosis-mediated stimulation of surface polysaccharide production and markedly compromises aerotolerance in C. jejuni. These results in this study provide novel insights into how an oxygen-sensitive microaerophilic pathogen survives in oxygen-rich environments by adapting its metabolism and physiology.

**IMPORTANCE** Oxygen-sensitive foodborne pathogens must withstand oxygen toxicity in aerobic environments during transmission to humans. C. jejuni is a major cause of gastroenteritis, accounting for 400 million to 500 million infection cases worldwide per year. As an obligate microaerophile, C. jejuni is sensitive to air-level oxygen. However, it has not been fully explained how this oxygen-sensitive zoonotic pathogen survives in aerobic environments and is transmitted to humans. Here, we show that under aerobic conditions, C. jejuni boosts its carbon metabolism to produce a thick layer of bacterial capsules, which in turn act as a protective barrier conferring aerotolerance. The new findings in this study improve our understanding of how oxygen-sensitive C. jejuni can survive in aerobic environments.

## INTRODUCTION

Campylobacter jejuni is a leading bacterial cause of foodborne illnesses worldwide (1, 2) and is the primary cause of Guillain-Barré syndrome, an acute and progressive neuromuscular paralysis (3, 4). Although C. jejuni is transmitted to humans mainly through the consumption of contaminated poultry meat (5), the wide distribution of C. jejuni in a range of animal species, including livestock, pets, and wildlife, and the environment makes human campylobacteriosis possible by direct contact with infected animals or exposure to contaminated environmental sources (5–7). As an obligate microaerophile, C. jejuni is sensitive to atmospheric levels of oxygen but still requires low (3 to 10%) levels of oxygen for growth (8, 9).

C. jejuni contains oxygen-sensitive metabolic proteins essential for survival, such as flavodoxin-dependent pyruvate:acceptor oxidoreductase (Por) and 2-oxoglutarate:acceptor oxidoreductase (Oor) (10). These oxygen-sensitive enzymes crucial for carbon metabolism contain iron-sulfur (Fe-S) clusters that are vulnerable to inactivation by reactive oxygen species (ROS) (10). Also, C. jejuni contains oxygen-labile l-serine dehydratase (SdaA), which plays a critical role in carbon catabolism and colonization of the gastrointestinal tract of chickens (10). Moreover, C. jejuni is unable to grow under strict anaerobic conditions (11), because C. jejuni possesses a single class I ribonucleotide reductase, which requires oxygen for the conversion of nucleotides to deoxynucleotides; thus, C. jejuni can not synthesize DNA without oxygen (8). Currently, it is not fully understood how C. jejuni survives during zoonotic transmission generally involving aerobic environments and causes approximately 400 million to 500 million diarrheal cases worldwide per year (1).

Bacterial tolerance to environmental stress significantly increases the risk of human infection by enabling foodborne pathogens to survive during transmission (12, 13). Through a comparative genomic fingerprinting analysis, we have demonstrated that C. jejuni strains tolerant to stress conditions present in food processing are more frequently implicated in human infections than stress-sensitive strains (14). Among various environmental stresses, oxygen toxicity under aerobic conditions is a common stressor that C. jejuni unavoidably encounters during the course of transmission from its animal hosts to humans, whether or not involving food contamination (15). For oxygen-sensitive pathogens, survival under aerobic conditions is a prerequisite to the initiation of infection (16–18). In our previous studies, we have shown that the levels of aerotolerance of C. jejuni isolates from clinical cases and food significantly vary and that C. jejuni strains with increased aerotolerance are highly prevalent in retail raw poultry products (14, 19–22). Moreover, aerotolerant C. jejuni can survive on poultry meat for an extended period compared to oxygen-sensitive C. jejuni (23). Whereas oxygen-sensitive C. jejuni strains lose viability on poultry meat rapidly within a few days, aerotolerant C. jejuni strains can survive ~2 to 4 times longer than oxygen-sensitive strains (23). Furthermore, aerotolerance has also been reported in Campylobacter coli, another important pathogenic species of Campylobacter (24, 25). Aerotolerant C. coli strain OR12 has increased peroxide stress tolerance, is motile under aerobic conditions, and can colonize chicken intestines (24). These data suggest that aerotolerance can directly affect food safety at both pre- and postharvest levels by increasing the survival of Campylobacter in food and the environment and by facilitating horizontal transmission among animal hosts.

Despite the importance of aerotolerance in food safety risks associated with Campylobacter, the molecular mechanisms of aerotolerance are largely unknown, particularly regarding the physiological features underpinning the survival of Campylobacter in aerobic environments. In this study, we demonstrate that under aerobic conditions, C. jejuni stimulates central carbon metabolism to develop a thick layer of bacterial capsules, which can act possibly as a permeability barrier protecting C. jejuni from excess oxygen under aerobic conditions.

## RESULTS

### Aerobiosis stimulates surface polysaccharide production in C. jejuni.

**FIG 1:**
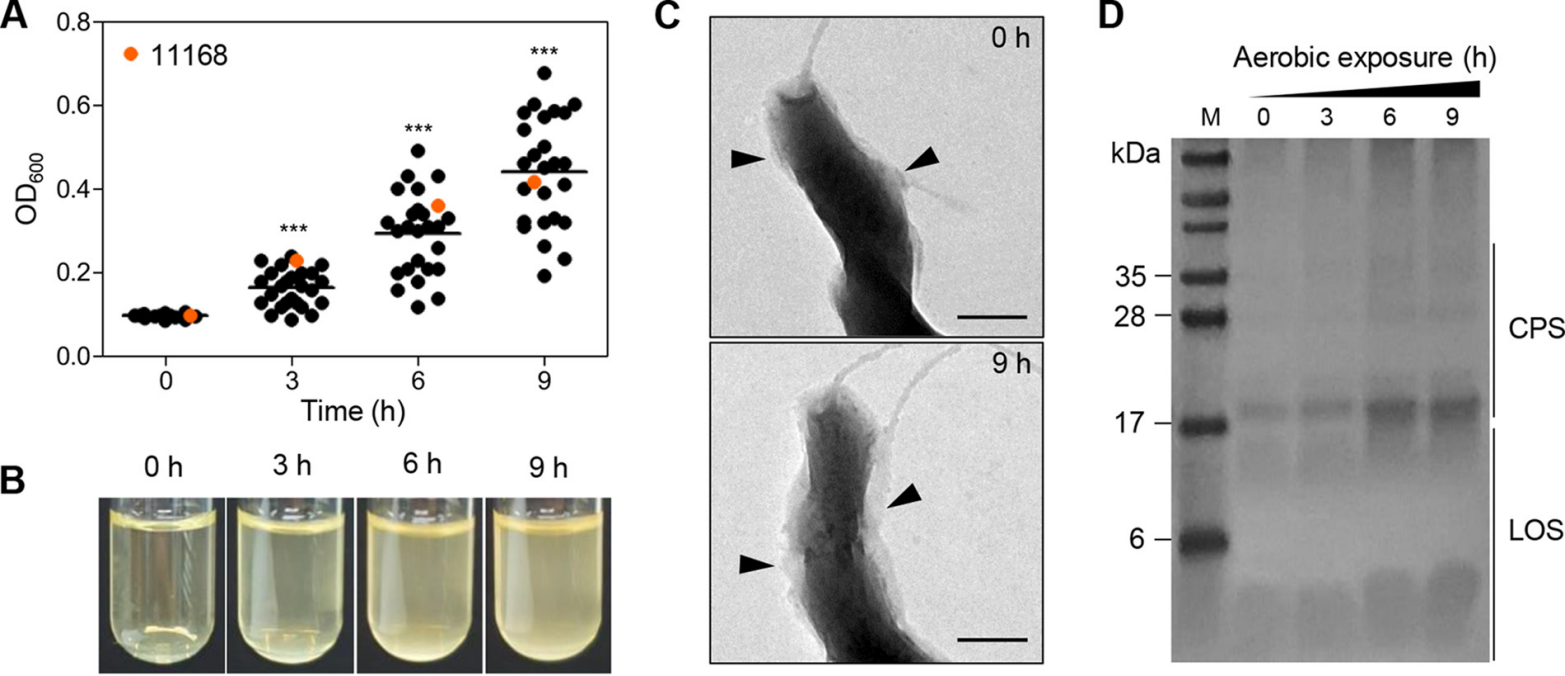
Stimulation of CPS and LOS synthesis by aerobiosis in C. jejuni. (A) The OD_600_ of C. jejuni NCTC 11168 and 25 C. jejuni isolates from retail chicken under aerobic conditions. C. jejuni strains were incubated with shaking at 200 rpm under aerobic conditions for 9 h. Solid horizontal lines indicate average OD_600_ values. Statistical significance was determined with Student’s *t* test compared to the OD_600_ of the previous time point, ***, *P < *0.001. (B) Bacterial culture images of C. jejuni NCTC 11168 over the time of exposure to aerobic conditions. (C) Transmission electron microscopic (TEM) images of C. jejuni NCTC 11168 before and after exposure to aerobic conditions for 9 h. Black triangles indicate surface polysaccharides stained with alcian blue. Scale bars = 200 nm. (D) Alcian blue staining of CPS and LOS in C. jejuni NCTC 11168 after exposure to aerobic conditions. M, marker.

We assessed the aerobic survival of C. jejuni NCTC 11168 and 25 C. jejuni chicken isolates, which were collected from retail poultry in our previous study (21). Interestingly, the optical density at 600 nm (OD_600_) of aerobic cultures of all the tested strains was markedly increased (Fig. 1A and B) despite the inability of C. jejuni to grow aerobically (see Fig. S1 in the supplemental material). Since the increases in OD_600_ were not corelated with the CFU in aerobic cultures, we hypothesized that C. jejuni might undergo alterations in bacterial morphology and/or surface structures under aerobic conditions, which may result in OD changes. To examine our hypothesis, transmission electron microscopy (TEM) was conducted to observe the morphology of C. jejuni cells from aerobic cultures in combination with alcian blue staining to visualize surface polysaccharides (26). C. jejuni NCTC 11168, the first whole-genome-sequenced strain of Campylobacter (27), was used to investigate the molecular mechanisms of aerotolerance in the rest of the study. Remarkably, aerobiosis led to the formation of a thick layer of bacterial capsules in C. jejuni (Fig. 1C). Alcian blue staining analyses confirmed that the production of capsular polysaccharide (CPS) and lipooligosaccharide (LOS) was increased over time after aerobiosis (Fig. 1D). These results suggest that under aerobic conditions, C. jejuni increases the production of surface polysaccharides, which can affect the measurement of OD_600_ under aerobic conditions.

### Disruption of surface polysaccharide integrity with EDTA decreases the optical density and compromises aerotolerance in C. jejuni.

**FIG 2:**
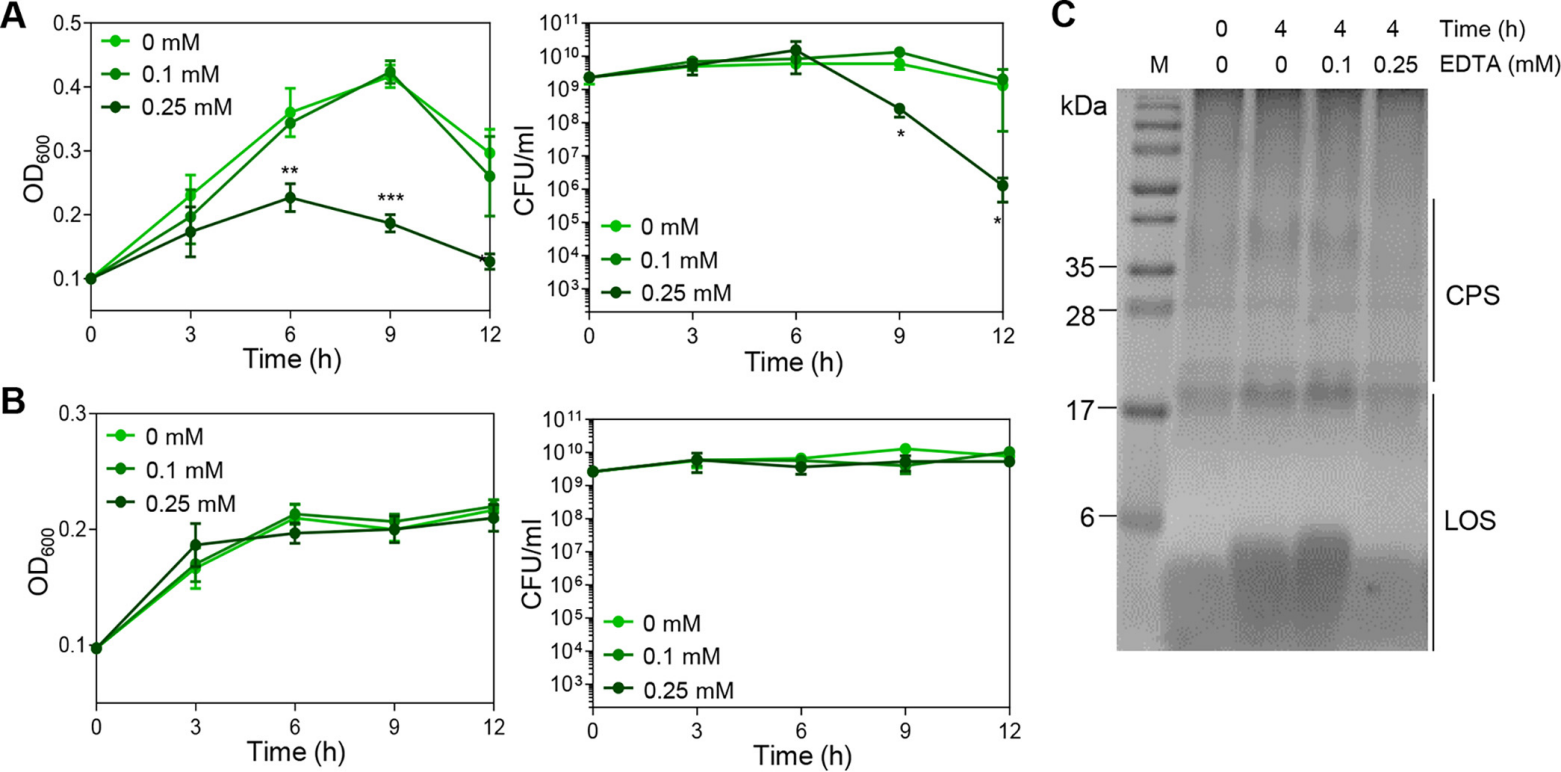
Compromised aerotolerance by disrupting the integrity of surface polysaccharides with EDTA. (A and B) The OD_600_ and CFU of C. jejuni NCTC 11168 under aerobic (A) and microaerobic (B) conditions in MH broth supplemented with different concentrations of EDTA. The data present the means and the standard errors of the mean (SEM) of the results of three independent experiments. Statistical significance was determined with Student’s *t* test compared to a nontreated sample at the same time point; *, *P < *0.05; **, *P < *0.01; ***, *P < *0.001. (C) Alcian blue staining images of CPS and LOS in C. jejuni NCTC 11168 according to exposure time under aerobic conditions in the presence and absence of EDTA. M, marker.

To evaluate whether OD_600_ increases in aerobic cultures are associated with surface polysaccharide synthesis, C. jejuni NCTC 11168 was subjected to aerobiosis in the presence of EDTA to disrupt the integrity of surface polysaccharides by chelating divalent ions. The bacterial surface of C. jejuni is decorated with CPS and LOS, a truncated version of lipopolysaccharide (LPS) lacking O-antigen (28). In Gram-negative bacteria, divalent ions are involved in maintaining the structural integrity of bacterial surfaces by cross-linking phosphate groups in the lipid anchor of CPS and the inner core of LPS (29–31). Notably, the supplementation of C. jejuni cultures with 0.25 mM EDTA significantly reduced the OD_600_ and CFU under aerobic conditions compared to a non-treated control (Fig. 2A). Consistent with a previous report (19), C. jejuni NCTC 11168 lost viability after exposure to aerobic stress for 24 h (data not shown). Under microaerobic conditions, 0.25 mM EDTA did not alter the OD_600_ and CFU (Fig. 2B), indicating that EDTA decreases the OD_600_ and CFU only in aerobic cultures. Furthermore, EDTA treatment depleted surface polysaccharides (Fig. S2) and decreased the abundance of CPS and LOS under aerobic conditions (Fig. 2C). Together, these results suggest that the stimulated production of surface polysaccharides by aerobiosis increases the OD_600_ and affects aerotolerance in C. jejuni.

### Both CPS and LOS are required for aerotolerance in C. jejuni.

**FIG 3:**
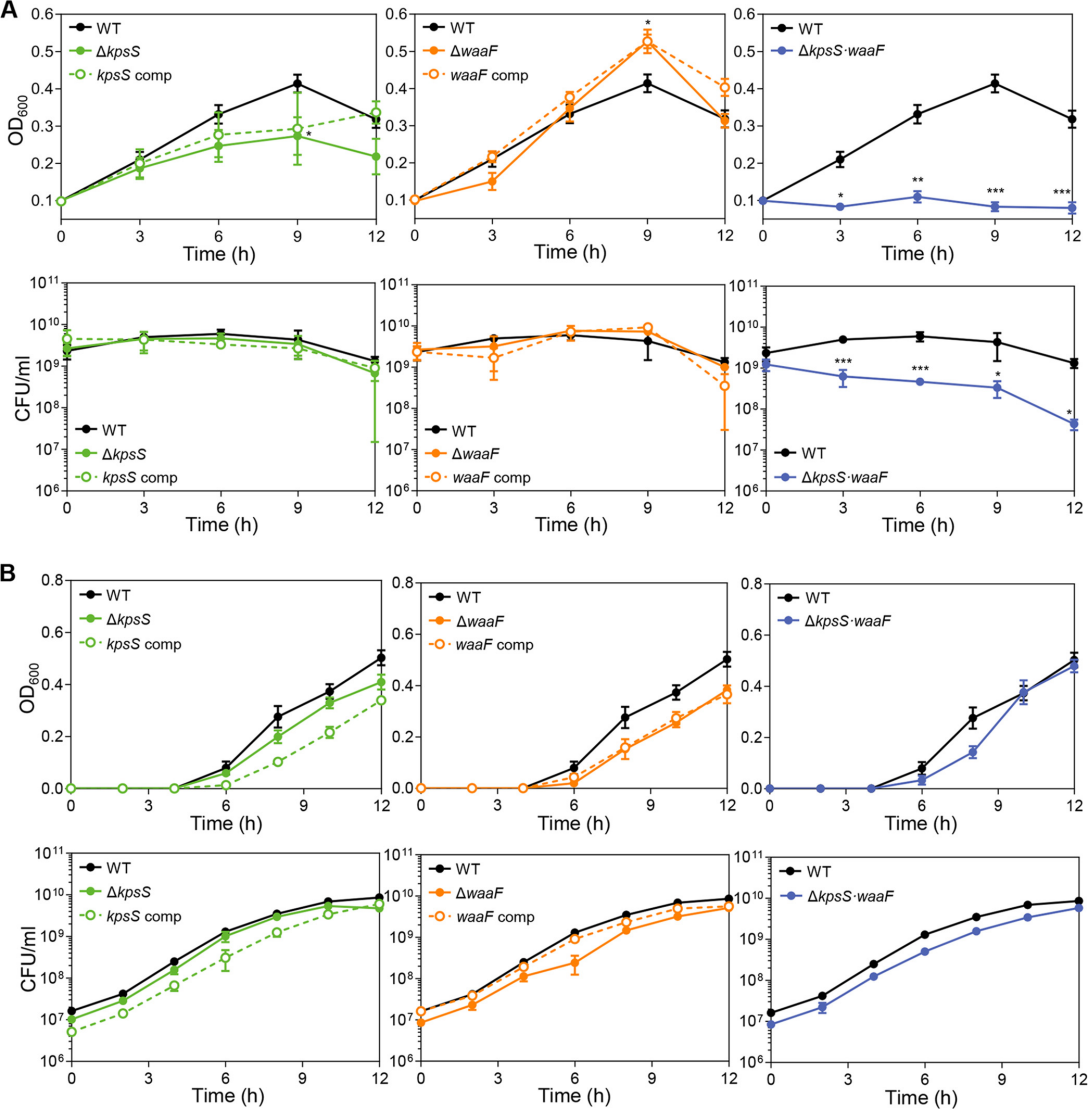
Compromised aerotolerance in mutants defective in CPS and LOS synthesis. The (A and B) OD_600_ and CFU of C. jejuni mutants defective in the synthesis of surface polysaccharides after aerobic (A) and microaerobic (B) cultivation with shaking at 200 rpm. Statistical significance was determined with Student’s *t* test compared to the values of the wild type (WT; C. jejuni NCTC 11168) at the same time point (*, *P < *0.05; **, *P < *0.01; ***; *P < *0.001). A Δ*kpsS* mutant (Δ*kpsS*), a *kpsS*-complemented strain (*kpsS* comp), a Δ*waaF* mutant (Δ*waaF*), a *waaF*-complemented strain (*waaF* comp), and a Δ*kpsS*/Δ*waaF* double mutant (Δ*kpsS·waaF*).

After examining the involvement of surface polysaccharides in aerotolerance with EDTA (Fig. 2), we validated this finding using the mutants defective in the production of surface polysaccharides. The synthesis of CPS and LOS is a complicated process involving a number of genes. A knockout mutation of a gene associated with the early step of CPS and LOS biosynthesis can lead to defects in CPS and LOS production. For this, we disrupted the synthesis of CPS and LOS by deleting *kpsS* and *waaF*, respectively (32–34). The *kpsS* gene encodes an enzyme responsible for the production of the 3-deoxy-d-manno-oct-2-ulosonic acid (Kdo) linker on the terminal lipid of CPS (35). A knockout mutation of *waaF*, which encodes the heptosyltransferase II enzyme, results in a truncation of the core oligosaccharide (32, 36). We measured the OD_600_ and viability of these mutants under aerobic conditions. The OD_600_ increase in the aerobic cultures of a CPS (Δ*kpsS*) mutant was less than that of the wild type (WT; Fig. 3A and Fig. S3). Aerobic exposure did not diminish the OD_600_ of the cultures of an LOS (Δ*waaF*) mutant but, rather, increased it (Fig. 3A and Fig. S3), presumably because the lack of LOS resulted in auto-agglutination of bacterial cells exposing hydrophobic lipid membranes (34, 37). A double knockout mutation of *kpsS* and *waaF* eliminated the OD_600_ increases under aerobic conditions (Fig. 3A and Fig. S3). However, the OD_600_ of the Δ*kpsS*/Δ*waaF* double mutant was comparable to that of the WT under microaerobic conditions (Fig. 3B). Remarkably, the Δ*kpsS*/Δ*waaF* double mutant showed a substantial defect in aerotolerance (Fig. 3A).

Genetic complementation in the Δ*kpsS* mutant and Δ*waaF* mutant resulted in the recovery of aerotolerance. The phenotype recovery in these mutants by complementation was partial under some conditions (Fig. 3A), presumably because the transcription of the complemented genes was driven by different promoters. For instance, *kpsS* exists in an operon, and its transcription is modulated by its operon promoter; however, *kpsS* transcription in the complementation strain is controlled by the constitutively expressing promoter of the antibiotic resistance cassette (38). Additionally, a strain complemented with both *kpsS* and *waaF* could not be constructed in the Δ*kpsS*/Δ*waaF* mutant because of the limited availability of antibiotic resistance markers in Campylobacter research. The two antibiotic-selective markers (i.e., kanamycin and tetracycline resistance markers) were already used to construct the Δ*kpsS*/Δ*waaF* double mutant, leaving only the chloramphenicol resistance marker available. Due to this technical issue, we could test phenotype recovery by complementing the Δ*kpsS*/Δ*waaF* double mutant only with a single gene, either *kpsS* or *waaF*. The complementation of the double mutant with a single gene partially restored the OD_600_ increases and CFU under aerobic conditions (Fig. S4). Nevertheless, these results from these knockout mutants and their complementation strains collectively confirm that the OD_600_ increase in the aerobic cultures of C. jejuni is associated with aerobiosis-mediated stimulation of surface polysaccharide production, which contributes to aerotolerance in C. jejuni.

### Carbon metabolism genes are up-regulated in C. jejuni under aerobic conditions.

**FIG 4:**
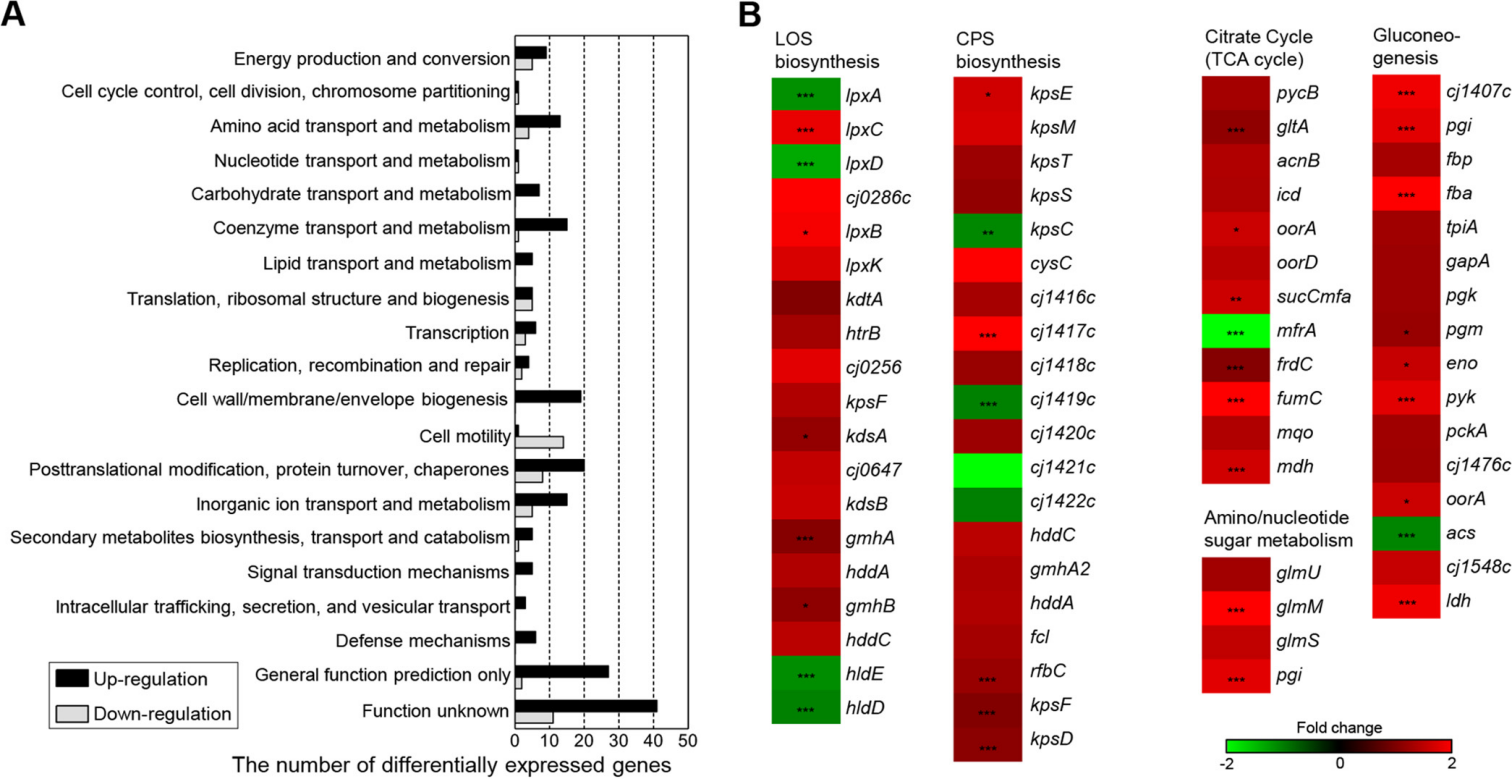
Transcriptomic changes in C. jejuni under aerobic conditions. (A) Differentially expressed genes in C. jejuni under aerobic conditions based on RNA-Seq. The fold change was determined by comparing the transcriptional levels between aerobic and microaerobic conditions. The black and gray bars indicate genes up-regulation and down-regulation, respectively, after aerobic exposure. (B) Heat maps of the genes associated with CPS and LOS biosynthesis and carbon metabolism under aerobic conditions. The heat maps were constructed with Gitools.

Next, we wondered whether aerobiosis may alter the transcription of genes of CPS and LOS synthesis. To answer this question, we performed transcriptome sequencing (RNA-Seq) to measure transcriptomic changes under aerobic conditions. The analysis revealed that the transcription of 16.5% (271/1,643) of the total genes of C. jejuni NCTC 11,168 was altered by aerobiosis compared to the transcriptomic profile of microaerobic cultures (Fig. 4A and Table S1). Among the differentially expressed genes under aerobic conditions, 208 of them were up-regulated, while 63 were down-regulated (Fig. 4A and Table S1). The genes associated with energy production and conversion, nutrient transport and metabolism, cell envelope biogenesis, posttranslational modification, protein turnover, chaperones, and signal transduction mechanisms were up-regulated under aerobic conditions, whereas the genes involved in cell motility were generally down-regulated (Fig. 4A and Table S1). Several oxidative stress defense genes, including *ahpC* (alkyl hydroperoxide reductase), *sodB* (superoxide dismutase), *cosR* (Campylobacter oxidative stress regulator), and *perR* (peroxide response regulator), were up-regulated in response to exposure to aerobic conditions (Table S1). Consistent with increases in the production of surface polysaccharides by aerobiosis, the genes related to CPS and LOS synthesis were up-regulated in aerobic cultures (Fig. 4B). Additionally, aerobiosis up-regulated genes encoding the enzymes of the TCA cycle and gluconeogenesis (Fig. 4B). These results show that aerobiosis up-regulates the genes of oxidative stress, CPS and LOS synthesis, and carbon metabolism in C. jejuni.

### Production of precursors for surface polysaccharides was increased under aerobic conditions.

**FIG 5:**
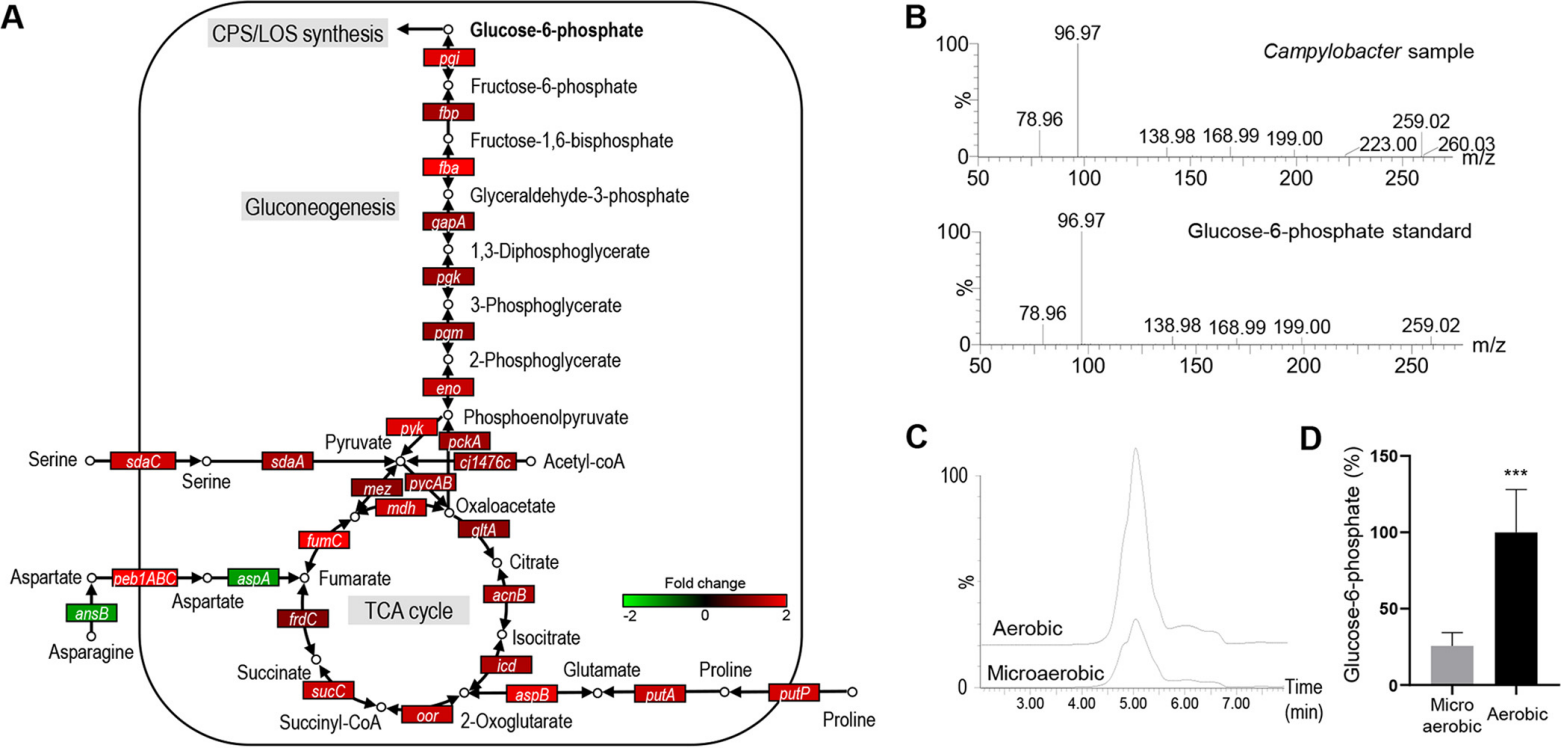
Increased production of precursors for surface polysaccharide synthesis under aerobic conditions. (A) Upregulation of the genes of the tricarboxylic acid (TCA) cycle and the Embden-Meyerhof-Parnas (EMP) pathway in C. jejuni NCTC 11168 under aerobic conditions. Fold change was determined by comparing the transcriptional levels between aerobic and microaerobic conditions. (B) Identification of glucose-6-phosphate in C. jejuni NCTC 11168 extract through comparison with the fragmentogram of its authentic standard. (C) Overlaid LC-MS chromatograph showing increased production of glucose-6-phosphate under aerobic conditions compared to microaerobic conditions. (D) Comparison of the levels of glucose-6-phosphate between aerobic and microaerobic cultures. The MS signals of glucose-6-phosphate in aerobic samples were arbitrarily set as 100%. Statistical significance was assessed with Student’s *t* test (***, *P < *0.001).

Aerobiosis upregulates the genes of central carbon metabolism (Fig. 4B). Given the inability of aerobic growth of C. jejuni, the purpose of boosting carbon metabolism under aerobic conditions is not likely to promote bacterial growth but possibly to produce precursors for the synthesis of surface polysaccharides. Due to the lack of the genes encoding glucokinase (Glk) and phosphofructokinase (Pfk) of the Embden-Meyerhof-Parnas (EMP) pathway (27), C. jejuni is unable to catabolize glucose and primarily relies on amino acids and the TCA cycle intermediates as carbon sources (39–42). Using the remaining EMP pathway enzymes, C. jejuni synthesizes glucose and EMP pathway intermediates through gluconeogenesis, which is the only mechanism for C. jejuni to produce precursors for surface polysaccharides (43). In particular, glucose-6-phosphate generated by gluconeogenesis is the ultimate EMP pathway intermediate required to produce UDP-*N*-acetylglucosamine (UDP-GlcNAc) and sedoheptulose-7-phosphate (Fig. S5) for the synthesis of surface polysaccharides (Fig. 5A). We compared the intracellular levels of glucose-6-phosphate between the aerobic and microaerobic cultures of C. jejuni and found that aerobiosis significantly increased the level of glucose-6-phosphate by 3.9-fold more than microaerobiosis (Fig. 5B to D). Taken together, these results suggest that alterations in carbohydrate metabolism by aerobiosis increase the level of glucose-6-phosphate to supply precursors for surface polysaccharide synthesis under aerobic conditions.

### The TCA cycle is involved in aerobiosis-mediated induction of surface polysaccharide synthesis and aerotolerance in C. jejuni.

**FIG 6:**
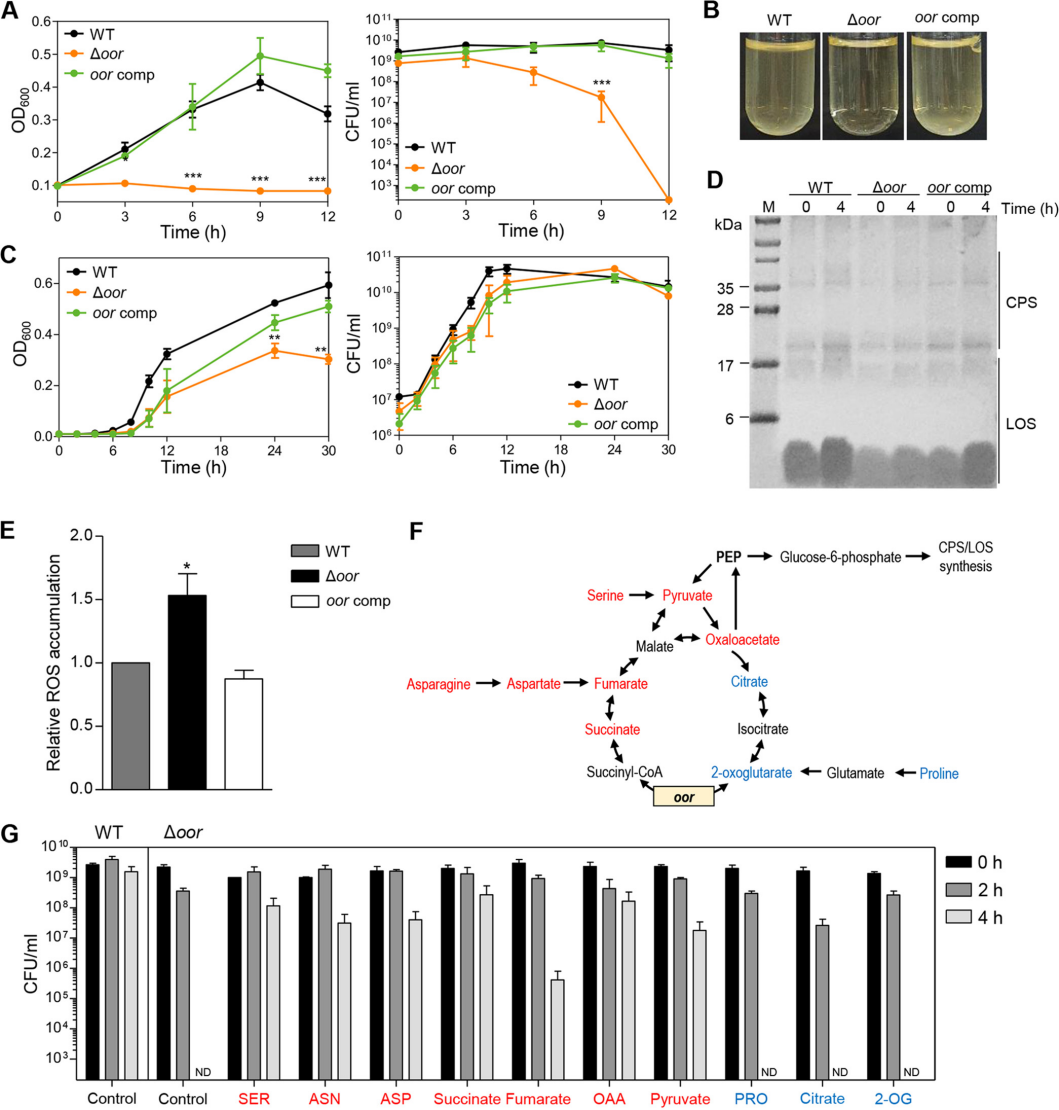
Defects in surface polysaccharide synthesis and aerotolerance in an Δ*oor* mutant. (A) The OD_600_ and CFU of the wild type (WT; C. jejuni NCTC 11168), an Δ*oor* mutant, and an *oor*-complemented strain in MH broth under aerobic conditions are shown. The data present the means and the standard errors of the mean (SEM) of the results of three experiments. Statistical significance was determined with Student’s *t* test compared to WT at the same time point (**, *P < *0.01; ***, *P* < 0.001). (B) Bacterial culture images of the WT, an Δ*oor* mutant, and an *oor*-complemented strain under aerobic conditions for 4 h. (C) The OD_600_ and CFU of microaerobic cultures of WT, an Δ*oor* mutant, and an *oor*-complemented strain in MH broth. The data present the means and SEM of the results of three experiments. Statistical significance was determined with Student’s *t* test compared to the WT at the same time point (**, *P < *0.01). (D) Surface polysaccharides under aerobic conditions in the WT, an Δ*oor* mutant, and an *oor*-complemented strain. The image shows alcian blue staining of CPS and LOS in the WT, an Δ*oor* mutant, and an *oor*-complemented strain before/after aerobiosis. M, marker. (E) Relative levels of the total ROS in the WT, an Δ*oor* mutant, and an *oor*-complemented strain after aerobiosis for 4 h. The level of the WT was set as 1. The data present the means and SEM of the results of six experiments. Statistical significance was determined by Student’s *t* test compared to the WT (*, *P < *0.05). (F) A simplified model of the TCA cycle and gluconeogenesis in C. jejuni. Carbon sources increasing aerotolerance in the Δ*oor* mutant are indicated in red, and those that did not are in blue. (G) Aerotolerance of WT (control) and an Δ*oor* mutant in the presence of amino acids and TCA cycle intermediates. The CFU levels of the WT and the Δ*oor* mutant were determined under aerobic conditions for 4 h with shaking (200 rpm) in MEMα with or without (control) supplemental carbon sources. SER, serine; ASN, asparagine; ASP, aspartate; OAA, oxaloacetate; PRO, proline; 2-OG, 2-oxoglutarate; ND, not detected. The data present the means and SEM of the results of at least three experiments. For all data except for F, an Δ*oor* mutant (Δ*oor*) and an *oor*-complemented strain (*oor* comp) were used.

Several genes encoding the enzymes of the TCA cycle were up-regulated under aerobic conditions (Fig. 5A). Amino acids taken up by C. jejuni are integrated into carbohydrate metabolism after enzymatic conversion to the TCA cycle intermediates, which is the major metabolic pathway to absorb carbon sources in C. jejuni (43). The TCA cycle is the essential metabolism that provides precursors to the EMP pathway for gluconeogenesis, which produces glucose-6-phosphate for the synthesis of surface polysaccharides (Fig. 5A). We wondered about the role of the TCA cycle in the stimulation of surface polysaccharide synthesis under aerobic conditions and aerotolerance in C. jejuni. Thus, we evaluated the association of the TCA cycle with aerobiosis-mediated induction of surface polysaccharide synthesis. As reported in previous studies (44, 45), we observed that aerobiosis up-regulated *oorDABC* (Fig. 5A) encoding 2-oxoglutarate: acceptor oxidoreductase (Oor) that decarboxylates 2-oxoglutarate to succinyl-CoA (46, 47). The increased transcription level of *oorDABC* under aerobic conditions was confirmed with quantitative real-time PCR (qRT-PCR) (Fig. S6). A knockout mutation of *oor* eliminated the aerobiosis-mediated induction of the OD_600_ increase and significantly reduced the viability of C. jejuni under aerobic conditions (Fig. 6A and B). As opposed to obvious changes in the OD_600_ under aerobic conditions (Fig. 6A), the disruption of *oor* slightly reduced the OD_600_ of C. jejuni cultures under microaerobic conditions (Fig. 6C). The complementation of the Δ*oor* mutant with an intact copy of *oor* did not restore the OD_600_ until 12 h but increased it close to the WT level after extended incubation under microaerobic conditions (Fig. 6C). Presumably, *oor* expression and/or regulation may differ between aerobic and microaerobic conditions. Consistently, the production of surface polysaccharides was not stimulated by aerobiosis in the Δ*oor* mutant (Fig. 6D and Fig. S7). Furthermore, the accumulation of total reactive oxygen species (ROS) was significantly increased in the Δ*oor* mutant under aerobic conditions compared to that in the WT (Fig. 6E), which accounts for compromised aerotolerance in the Δ*oor* mutant.

The association of the TCA cycle with aerotolerance was evaluated in an alternative way by chemically complementing the Δ*oor* mutant with TCA cycle intermediates and amino acids. Notably, the aerotolerance of the Δ*oor* mutant was restored when the Δ*oor* mutant was cultured on the minimum essential medium (MEM) α supplemented with various amino acids and TCA cycle intermediates, which can be converted to phosphoenolpyruvate (PEP; Fig. 6F). Some carbon sources, such as serine, asparagine, aspartate, succinate, oxaloacetate, and pyruvate, restored aerotolerance to levels close to that of the WT (Fig. 6G). These compounds are commonly involved in steps following the process mediated by Oor (Fig. 6F and G). However, aerotolerance was not restored by supplementing with proline, citrate, and 2-oxoglutarate, which are associated with the TCA cycle steps preceding the Oor step (Fig. 6F and G). These data suggest that the stimulated TCA cycle under aerobic conditions is required to facilitate surface polysaccharide synthesis and aerotolerance in C. jejuni.

### Oxidative stress is increased in mutants defective in surface polysaccharide production under aerobic conditions.

**FIG 7:**
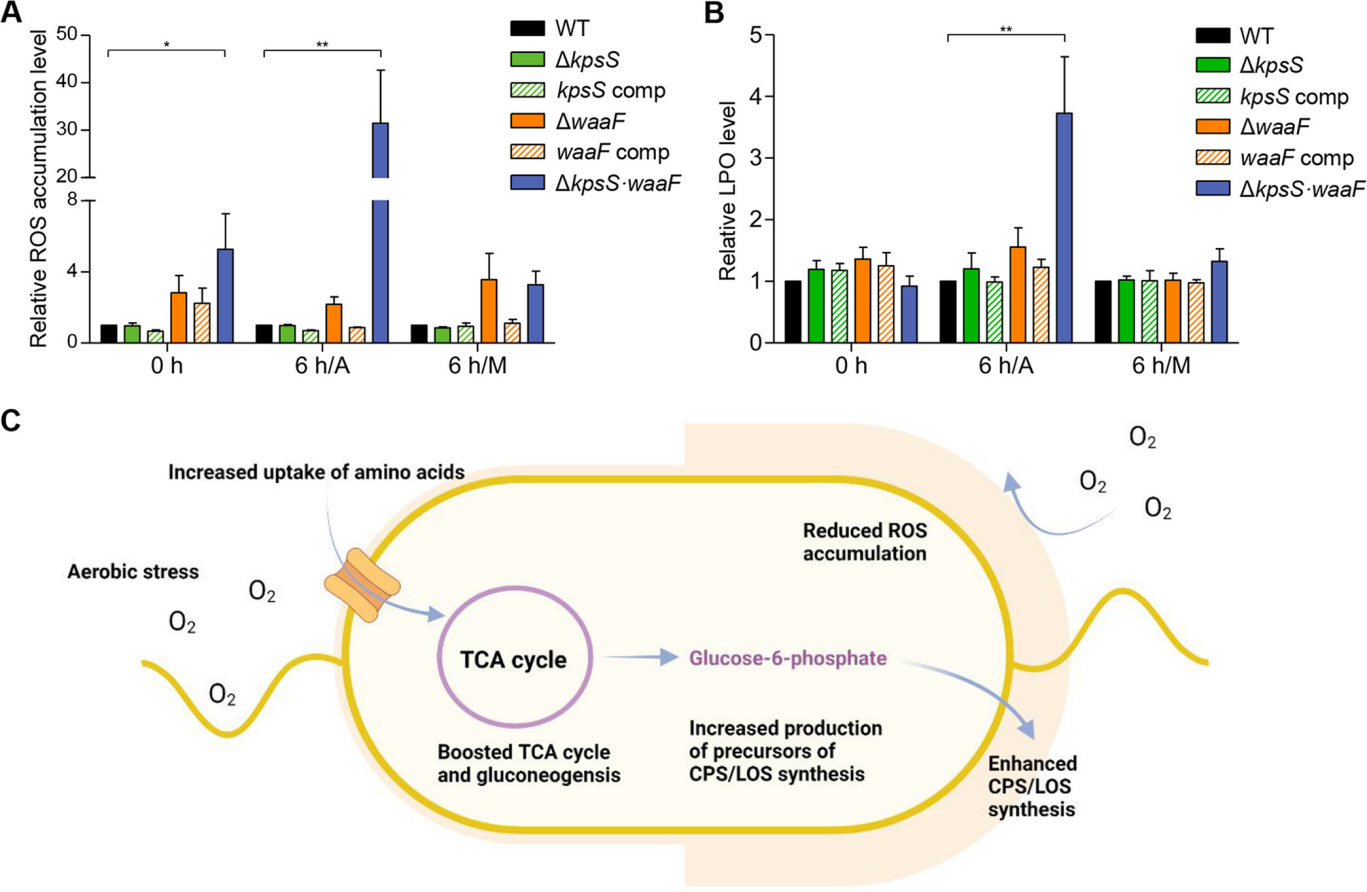
Contribution of CPS and LOS to aerotolerance by alleviating oxidative stress. (A and B) Relative levels of the total ROS accumulation (A) and lipoperoxide (LPO) (B) of C. jejuni strains before/after exposure to aerobic and microaerobic conditions for 6 h. The levels of ROS and LPO in the WT were set as 1. A, aerobic conditions; M, microaerobic conditions. Data for a Δ*kpsS* mutant (Δ*kpsS*), a *kpsS*-complemented strain (*kpsS* comp), a Δ*waaF* mutant (Δ*waaF*), a *waaF*-complemented strain (*waaF* comp), and a Δ*kpsS*/Δ*waaF* double mutant (Δ*kpsS waaF*) are shown. The data present the means and the standard errors of the mean (SEM) of the results of three experiments. Significance was assessed using one-way ANOVA (*, *P < *0.05; **, *P* < 0.01; ns; not significant). (C) Stimulation of carbon metabolism and surface polysaccharide synthesis under aerobic conditions protects C. jejuni from excess oxygen in aerobic environments.

The disruption of the TCA cycle reduces surface polysaccharide synthesis and increases oxidative stress (Fig. 6D and E). This indicates that the presence of surface polysaccharides can increase aerotolerance by alleviating oxidative stress. To examine this possibility, we measured the levels of total ROS and lipoperoxide (LPO) in mutants defective in the biosynthesis of CPS (Δ*kpsS*), LOS (Δ*waaF*), and both (Δ*kpsS* and Δ*waaF*). Compared to a CPS (Δ*kpsS*) mutant, an LOS (Δ*waaF*) mutant showed increased levels of total ROS under aerobic and microaerobic conditions (Fig. 7A). Notably, ROS and LPO were significantly accumulated in the Δ*kpsS*/Δ*waaF* double mutant under aerobic conditions (Fig. 7A and B). Complementation of the Δ*kpsS*/Δ*waaF* double mutant with either *kpsS* or *waaF* partially decreased the level of ROS accumulation under aerobic conditions and reduced it to the WT level under microaerobic conditions (Fig. S8), indicating that CPS and LOS are required to alleviate oxidative stress mainly under aerobic conditions. These results suggest that the presence of CPS and LOS confers aerotolerance by protecting C. jejuni from oxidative damage under aerobic conditions.

## DISCUSSION

Aerotolerance is the critical mechanism for sustaining the viability of oxygen-sensitive C. jejuni in aerobic environments (48, 49). The aerobic survival of C. jejuni can be mediated by other mechanisms, such as biofilm development and the viable-but-non-culturable state; however, these survival mechanisms generally necessitate extensive physiological changes in C. jejuni, such as the physiological transition from planktonic to sessile lifestyle and from a culturable to nonculturable state (50–52). Regarding the molecular mechanism of aerotolerance, oxidative stress responses involving the ROS-detoxification enzymes, particularly alkyl hydroperoxide reductase (AhpC), are the key determinant of aerotolerance in C. jejuni (48, 49). In line with this, C. jejuni responds to oxidative stress differentially depending on the strain, which appears to determine whether a C. jejuni strain is oxygen sensitive or aerotolerant (22, 24, 44). Previously, we measured the activities of catalase and superoxide dismutase in 70 C. jejuni isolates from retail chicken and discovered that aerotolerant C. jejuni strains were more tolerant to peroxide and superoxide stress than oxygen-sensitive strains (22). C. jejuni strain Bf, an aerotolerant strain isolated from a clinical case, exhibits increased tolerance to oxidative stress and can better survive in the presence of oxidants compared to a reference strain (44). In addition, aerotolerant C. coli strains are more frequently isolated from retail food products than aerotolerant C. jejuni (25). Interestingly, the prevalence of aerotolerant C. coli is associated with the presence of catalase-like protein in C. coli (25). Other than the classic aerotolerance mechanism driven by oxidative stress responses, little information is available about the physiological features contributing to aerotolerance in C. jejuni. The data in this study demonstrate that under aerobic conditions, C. jejuni boosts carbon metabolism to increase amino acid uptake, stimulates the production of surface polysaccharides, and develops a thick layer of surface polysaccharides, which possibly acts as a permeability barrier to protect C. jejuni from excess oxygen in aerobic environments (Fig. 7C).

Surface polysaccharides are a frontline barrier protecting Gram-negative bacteria from the surrounding environment, playing various roles in host colonization (43, 53), bacteriophage infection (54), biofilm formation (55), serum resistance (56, 57), and antibiotic resistance (34). In particular, LPS makes the bacterial outer membrane impermeable to hydrophobic compounds (58–60), and so does LOS in C. jejuni (34). Oxygen, as a small nonpolar molecule, can freely diffuse across biological membranes (61, 62). However, the hydrophilic polar headgroup regions of phospholipids are barriers to oxygen permeation through membranes (63, 64). Thus, hydrophilic surface polysaccharide layers can limit the access of oxygen to the membrane of C. jejuni. Notably, C. jejuni thickens surface capsules under aerobic conditions, not microaerobic conditions, suggesting that the stimulation of surface polysaccharide synthesis is a unique survival mechanism to sustain the viability of C. jejuni under aerobic conditions. Similar findings have been reported in other bacteria. The nitrogen-fixing bacterium Azotobacter vinelandii produces thick alginate capsules on the bacterial surface as a barrier for oxygen transfer into the cell, which helps protect the O_2_-sensitive nitrogenase from inactivation (65). The formation of thick surface capsules appears to be a novel survival mechanism protecting oxygen-sensitive bacteria from aerobic stress.

Since polysaccharides are a predominant component of bacterial biofilms (66), the enhancement of surface polysaccharide production by aerobiosis can be an environmental factor enhancing biofilm development in C. jejuni. In our previous study, we showed that oxidative stress promotes the biofilm formation of C. jejuni (15, 67). Increased production of polysaccharides under aerobic conditions can enhance biofilm formation, possibly facilitating transition from a planktonic to sessile state. However, the validation of this hypothesis requires further investigation.

Previous studies also demonstrate the association of carbon metabolism with aerotolerance in Campylobacter. Proteomics and transcriptomics analyses of C. jejuni have shown that aerobiosis increases the abundance of several amino acid transporters and TCA cycle enzymes, including Oor (45). Similarly, aerobiosis of the aerotolerant C. jejuni strain Bf overexpresses the proteins of the TCA cycle and amino acid uptake in addition to the enzymes of oxidative stress responses compared to the proteomics profile of microaerobic conditions (44). Whole-genome sequencing of C. coli strain OR12 shows that this aerotolerant strain has insertional or deletional mutations in genes related to carbohydrate metabolism, such as the b subunit of pyruvate carboxylase (24). These studies show that carbohydrate metabolism genes are up-regulated under aerobic conditions. However, little was known about why aerobiosis activates carbon metabolism in C. jejuni, although this obligate microaerophile is unable to grow aerobically. The findings in this study demonstrate that carbon metabolism through the TCA cycle plays a critical role in the aerotolerance of C. jejuni by producing precursors for surface polysaccharide synthesis.

Oxygen-sensitive bacteria are generally equipped with unique aerotolerance mechanisms unseen in aerobic bacteria to protect from oxygen toxicity in oxygen-rich environments (17). For instance, aerobic bacteria use superoxide dismutase, which catalyzes the dismutation of superoxide into molecular oxygen. To avoid the enzymatic formation of oxygen by superoxide dismutase, anaerobes use superoxide reductases that reduce superoxide to hydrogen peroxide, which is converted to water by peroxidases (68, 69). Although the presence of superoxide reductase has been reported only in some microaerophiles, such as Treponema pallidum (70), it is not a general aerotolerance mechanism in microaerophiles. Unlike aerobes, microaerophiles must maintain the physiological balance between oxygen sensitivity and the requirement to respire with low levels of oxygen (16, 18). Based on the distribution of high-affinity terminal oxidases active at low oxygen concentrations, bacteria with the capability of microaerobic respiration are widespread in nature (18). However, little attention has been paid to molecular mechanisms whereby microaerophiles sustain viability in aerobic environments. Our study first demonstrates that the development of a thick layer of surface polysaccharides induced by aerobiosis protects the obligate microaerophilic bacterium C. jejuni from excess oxygen in aerobic environments. Since aerotolerance involves various cellular functions, such as ROS detoxification, respiration, and carbon metabolisms, our findings may provide only one mechanism underlying aerotolerance. However, our work overall expands our understanding of how an oxygen-sensitive microaerophilic pathogen sustains viability in oxygen-rich environments by adapting bacterial metabolism and physiology.

## MATERIALS AND METHODS

### Bacterial strains and growth conditions.

C. jejuni NCTC 11168 and 25 C. jejuni isolates from retail raw chicken in our previous study were used in this study (21). The strain was grown on Mueller-Hinton (MH) media (Oxoid, UK) at 42°C under microaerobic conditions (5% O_2_, 10% CO_2_, 85% N_2_). When needed, MH media were supplemented with kanamycin (50 μg/mL), chloramphenicol (12.5 μg/mL), and tetracycline (5 μg/mL). Escherichia coli DH5α was grown at 37°C on Luria-Bertani (LB) media (Difco, USA), which were occasionally supplemented with carbenicillin (100 μg/mL), kanamycin (50 μg/mL), tetracycline (5 μg/mL), and chloramphenicol (12.5 μg/mL), where required.

### Aerotolerance test.

The aerotolerance test was performed as described previously (19). Briefly, overnight cultures of C. jejuni on MH agar plates at 42°C were suspended in MH broth or MEMα medium to an OD_600_ of 0.1. The bacterial suspension (3 mL) in a 19-mL glass culture tube (catalog [cat.] no. T16-55-337; DWK Life Sciences, Germany) was incubated at 42°C with shaking (200 rpm) under aerobic conditions. Samples were taken at 2- or 3-h intervals for serial dilution and bacterial counting. To disrupt the cross-links of surface polysaccharides, EDTA was used at different concentrations (0.1 or 0.25 mM). MEMα was supplemented with serine, asparagine, aspartate, succinate, fumarate, oxaloacetate, pyruvate, proline, citrate, or 2-oxoglutarate to a final concentration of 20 mM to evaluate the effects of carbon sources on the restoration of aerotolerance in an Δ*oor* mutant.

### TEM analysis.

TEM using alcian blue staining was performed as described previously (71). Briefly, C. jejuni was fixed in ice-cold 2.5% glutaraldehyde and 1% paraformaldehyde in 0.1 M cacodylate buffer, and left overnight at room temperature with gentle inversion. After centrifugation at 10,000 × *g* for 5 min, C. jejuni strains were positively stained with saturated alcian blue solution. Samples were loaded on Formvar/carbon copper grids (200 mesh), and morphology was examined with an energy-filtering transmission microscope (EF-TEM; Libra 120, Germany) at a voltage of 120 kV.

### Detection of LOS and CPS by alcian blue staining.

CPS and LOS were detected with Alcian blue as described previously (26). Briefly, overnight cultures of C. jejuni strains on MH agar were suspended in MH broth or MEMα to an OD_600_ of 0.1. After exposure to either microaerobic or aerobic conditions, bacterial cultures were centrifuged at 10,000 × *g* for 5 min (MH broth) or 15,000 × *g* for 10 min (MEMα medium). Pellets were resuspended in lysis buffer (62.5 mM Tris-HCl, pH 6.8, 2% SDS, 10% glycerol, and 0.05% bromophenol blue) and boiled for 10 min. After centrifugation at 10,000 × *g* for 5 min, supernatants were mixed with proteinase K (final concentration of 1 mg/mL; Sigma, USA). The samples were incubated at 50°C for 1 h and then fractionated by SDS-PAGE. CPS and LOS were visualized with alcian blue staining (0.1% alcian blue dissolved in 40% ethanol/5% acetic acid).

### Growth experiments.

Overnight cultures of C. jejuni strains were harvested from MH agar plates and suspended in MH broth to an OD_600_ of 0.001 or 0.1. Cultures (20 mL) in 100-mL flasks were incubated at 42°C with shaking under microaerobic conditions. Samples were taken at 2- or 3-h intervals, and the OD_600_ was measured in an Ultrospec 2000 spectrophotometer (Amersham Pharmacia Biotech, USA) or serially diluted in phosphate-buffered saline (PBS). The suspension was serially diluted and plated on MH agar to enumerate CFU. All experiments were repeated three times.

### Construction of C. jejuni mutants and complemented strains.

For the construction of an Δ*oor* mutant, the gene with a flanking region was amplified by PCR using oor-SalI-F and oor-BamHI-R primers (Table S2). After restriction digestion of PCR products and pUC19, the PCR product was ligated to pUC19. pUC19::*oor* was inverse PCR-amplified with oor-inverse-F and oor-inverse-R primers (Table S2) and ligated with a kanamycin resistance cassette which amplified from pMW10 using Kan-F and Kan-R primers (Table S2). The suicide plasmid was transferred to C. jejuni NCTC 11168 by electroporation. In addition, a chromosomal integration method was used to construct an *oor*-complemented strain (38). The *oor* gene was amplified by PCR using oor-compl-F and oor-compl-R primers (Table S2) and digested by XbaI. After ligation with pFMBcomCM (72), the complementation plasmid was introduced to the *oor* mutant.

The *kpsS* (CPS) and *waaF* (LOS) single and double mutants were constructed previously (34). To construct *kpsS*- and *waaF*-complemented strains, the genes were amplified with PCR using kpsS-compl-F, kpsS-compl-R primers or waaF-compl-F, waaF-compl-R primers, respectively (Table S2). The PCR product was digested by XbaI and ligated with pFMBcomCM (72). The complementation plasmid was introduced to a Δ*kpsS* mutant or a Δ*waaF* mutant, and the transformants were selected by growing on MH agar plates supplemented with chloramphenicol (12.5 μg/mL). The single-gene (*kpsS* or *waaF*)-complemented double mutant was constructed by natural transformation with the genomic DNA of the *kpsS*- or *waaF*-complemented strains. The double mutant was grown overnight on MH agar, and the genomic DNA of the *kpsS*- or *waaF*-complemented strains was added to the culture. The C. jejuni strains were further grown for 5 h and plated on MH agar plates supplemented with kanamycin (50 μg/mL), tetracycline (5 μg/mL), and chloramphenicol (12.5 μg/mL).

The Δ*ahpC* mutant, *ahpC*-complemented strain, Δ*katA* mutant, *katA*-complemented strain, Δ*sodB* mutant, and *sodB*-complemented strain were constructed previously (72, 73).

### Total RNA extraction, RNA sequencing, and analysis.

To prepare bacterial total RNA, overnight cultures of C. jejuni on MH agar were harvested and suspended in MH broth to an OD_600_ of 0.1. Bacterial suspension (6 mL) in a 19-mL glass culture tube was incubated for 3 h with shaking under microaerobic conditions, and cultures were equally divided for additional cultivation at 42°C for 3 h with shaking under microaerobic and aerobic conditions (3 mL culture in a 19-mL glass culture tube). Bacterial cultures (μL) were treated with 5% ice-cold phenol-ethanol solution, and total bacterial RNAs were isolated using the RNeasy minikit (Qiagen, Germany) according to the manufacturer’s instructions. The quantity and quality of total RNA samples were examined using a NanoPhotometer N60 (Implen, USA), and two biological replicate RNA samples were submitted to Macrogen (Seoul, Republic of Korea) for RNA sequencing. Before sequencing, the quality and quantity of total RNA were rechecked using an Agilent Technologies 2100 Bioanalyzer with an RNA integrity number (RIN) value larger than 7. After mRNA-Seq library construction using the Illumina TruSeq RNA sample preparation kit v.2 (Illumina, USA), RNA-Seq was performed by two runs with an Illumina NovaSeq 6000 instrument to generate paired-end reads of around 101 bp in length. The expression level of each gene was normalized by calculating the reads per kilobase per million mapped reads (RPKM) using CLC Workbench. Fold change was defined as RPKM_aerobic conditions_/RPKM_microaerobic conditions_. The differentially expressed genes (DEGs; fold change ≥2 or ≤−2; *P < *0.05) were filtered and visualized using the Gitools.

### Quantitative real-time PCR (qRT-PCR).

The extraction of total RNA is described above. Using extracted RNA samples, cDNA was synthesized with cDNA EcoDry premix (Clontech, USA). The synthesized cDNA was mixed with 2 × iQ SYBR green supermix (Bio-Rad, USA) and 0.3 μM each primer in a reaction volume of 20 μL. All qRT-PCR primer sets used in this study are listed in Table S2. qRT-PCRs were performed using the CFX Connect real-time PCR detection system (Bio-Rad, USA). The cycling parameters were as follows: 95°C for 5 min; 39 cycles at 95°C for 15 s, 55°C for 15 s, 72°C for 30 s; 72°C for 7 min.

### Analysis of glucose-6-phosphate.

The extraction and the liquid chromatography-mass spectrometry (LC-MS) analysis of glucose-6-phosphate in C. jejuni were conducted according to a previously described protocol (74). C. jejuni NCTC 11168 was cultured on an MH agar plate overnight and resuspended in 200 mL of fresh MH broth to an OD_600_ of 0.08. The C. jejuni suspension was grown at 42°C microaerobically with shaking (200 rpm) for 4 h and was divided into two equal volumes. One volume was cultured at 42°C microaerobically with shaking (200 rpm) for 3 h, and the other was exposed to aerobic conditions with shaking (200 rpm) at 42°C for 3 h. C. jejuni was harvested with centrifugation at 3,000 × *g* at 4°C for 10 min and suspended in 0.5 mL of a methanol solution containing 1 μM sulfadimethoxine as the internal standard, sonicated for 15 s using an ultrasonic processor, and then mixed with 0.4 mL of water and 0.5 mL of chloroform for phase separation. After centrifugation at 13,000 × *g* at 4°C for 10 min, the aqueous phase was transferred to a fresh 1.5-mL tube and stored at −80°C prior to the analysis. For the LC-MS analysis, 5 μL of the extracted aqueous phase was injected into an Acquity ultraperformance liquid chromatography system (Waters, Milford, MA, USA). Separation was achieved in a 10-min run at a flow rate of 0.5 mL/min in a BEH amide column. The mobile phase used a gradient ranging from 99.5% aqueous Acetonitrile (ACN) containing 0.1% formic acid to 50% water. The LC eluent was then introduced into a Xevo-G2-S quadrupole time-of-flight mass spectrometer (Waters) for accurate mass measurement and ion counting. The accuracy of the MS was monitored by the intermittent injection of leucine encephalin ([M-H]– = *m/z* 554.2615). The capillary voltage and cone voltage for electrospray ionization (ESI) were maintained at −3 kV and −35 V for negative-mode detection, respectively. The source temperature and desolvation temperature were set at 120°C and 350°C, respectively. Nitrogen was used as both cone gas (50 L/h) and desolvation gas (600 L/h), and argon was used as collision gas. Structural information of glucose-6-phosphate was obtained by tandem MS (MS/MS) fragmentation with collision energies ranging from 15 to 50 eV, in comparison with an authentic standard.

### Measurement of total ROS.

The level of total ROS accumulation in C. jejuni was measured using CM-H_2_DCFDA (Life Technologies, USA). Briefly, overnight cultures of C. jejuni strains on MH agar were suspended in MH broth to an OD_600_ of 0.1. Bacterial suspension (3 mL) in a 19-mL glass culture tube was incubated at 42°C with shaking (200 rpm) under aerobic and microaerobic conditions for 6 h. After exposure to each condition, bacterial cultures were centrifuged at 10,000 × *g* for 5 min and washed twice with PBS (pH 7.4). Then, the pellet was resuspended with PBS and treated CM-H_2_DCFDA at a final concentration of 10 μM for 30 min. Fluorescence was measured with a SpectraMax i3 platform (Molecular Devices, USA). To normalize the ROS level, protein concentrations of each sample were measured with a Bradford assay (Bio-Rad, USA).

### LPO assay.

LPO levels were measured using a commercial kit (Cayman Chemical Co., USA) according to the manufacturer’s instructions. Briefly, overnight cultures of C. jejuni strains on MH agar were suspended in MH broth to an OD_600_ of 0.1. The bacterial suspension (3 mL) in a 19-mL glass culture tube was incubated at 42°C with shaking (200 rpm) under aerobic and microaerobic conditions for 6 h. After exposure to each condition, bacterial cultures were centrifuged at 10,000 × *g* for 5 min. LPOs were extracted with chloroform and methanol and mixed with the Chromogen reagent. After incubation at room temperature for 5 min, the OD at 500 nm was measured with a SpectraMax i3 platform (Molecular Devices, USA). A standard curve was generated with 13-hydroperoxy-octadecadienoic acid. The results were normalized with the protein concentration of each sample that was measured with the Bradford assay.

### Statistical analysis.

The data present the means and the standard errors of the mean (SEM) of the results of independent experiments. Statistical significance was evaluated with Student’s *t* test or one-way analysis of variance (ANOVA) using Prism version 5.01 (GraphPad Software, Inc., San Diego, CA, USA). *P* values of <0.05 were considered statistically significant.

### Data availability.

All RNA-Seq reads generated in this study were deposited in the Gene Expression Omnibus database (accession no. SRR17344685).
