# Supplementary material for: Stimulation of Surface Polysaccharide Production under Aerobic Conditions Confers Aerotolerance in Campylobacter jejuni
Source: Microbiol Spectr. 2023 Feb 14;11(2):e03761-22. doi: 10.1128/spectrum.03761-22 (PMC10100837; doi:10.1128/spectrum.03761-22)
Supplement: Supplemental file 1 — Supplemental material. Download spectrum.03761-22-s0002.pdf, PDF file, 0.5 MB [file spectrum.03761-22-s0002.pdf]

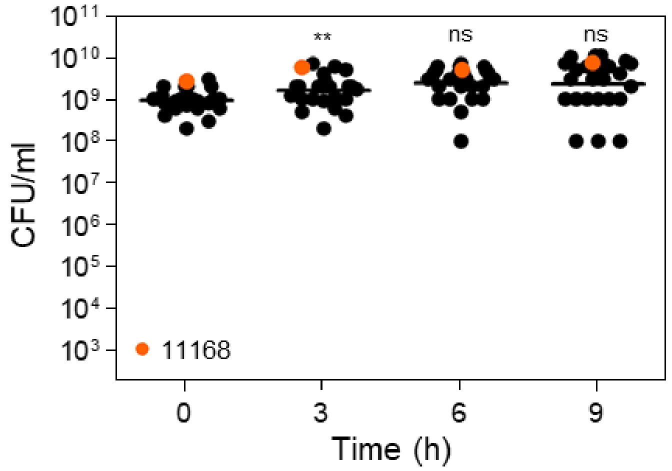

Fig. S1. The CFU values of 26 strains of *C. jejuni* under aerobic conditions. *C. jejuni* strains were incubated with shaking at 200 rpm under aerobic conditions for 9 h. Solid horizontal lines indicate average CFUs. Statistical significance was determined with Student's *t*-test compared to the CFU values of the previous time point. $^{**}P < 0.01$, ns; not significant.

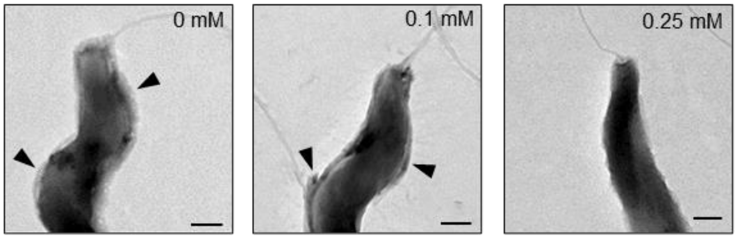

Fig. S2. Transmission electron microscopic (TEM) images of *C. jejuni* after exposure to aerobic conditions in the presence or absence of EDTA. *C. jejuni* NCTC 11168 was incubated with shaking at 200 rpm under aerobic conditions for 4 h with or without EDTA. Black triangles indicate surface polysaccharides stained with Alcian blue. Scale bars represent 200 nm.

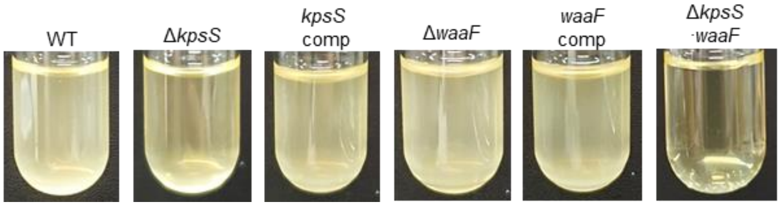

Fig. S3. Bacterial culture images of *C. jejuni* wild type (WT) and mutants defective in surface polysaccharide synthesis under aerobic conditions after 9 h. A Δ*kpsS* mutant (Δ*kpsS*), a *kpsS*-complemented strain (*kpsS* comp), a Δ*waaF* mutant (Δ*waaF*), a *waaF*-complemented strain (*waaF* comp), and a Δ*kpsS*/Δ*waaF* double mutant (Δ*kpsS*·*waaF*).

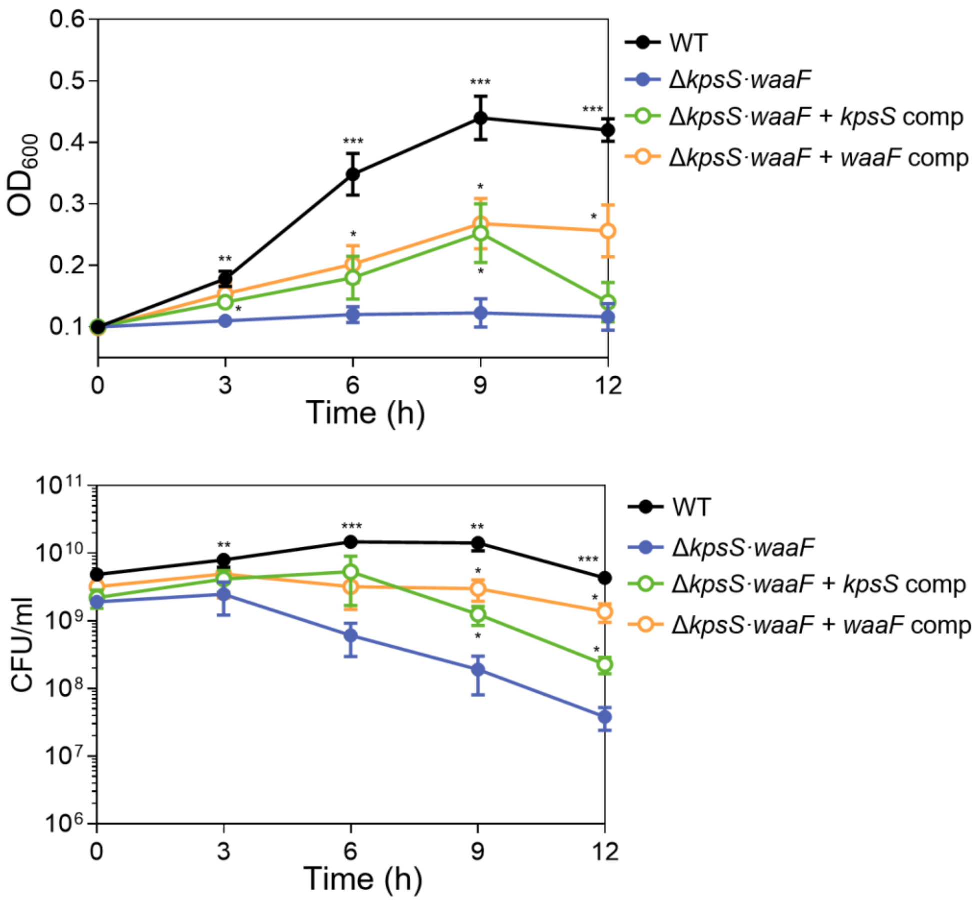

Fig. S4. Aerotolerance recovery in the Δ*kpsS*/Δ*waaF* double mutant by partial complementation with either *kpsS* or *waaF*. The OD_{600} and CFU of the *C. jejuni* strains after aerobic cultivation with shaking at 200 rpm. The data present the means and the standard errors of the mean (SEM) of the results of five experiments. Statistical significance was determined with Student's *t*-test compared to the values of the double mutant (Δ*kpsS·waaF*) at the same time point ($^{*}P < 0.05$, $^{**}P < 0.01$, $^{***}P < 0.001$). A wild type (WT), a Δ*kpsS*/Δ*waaF* double mutant (Δ*kpsS·waaF*), a *kpsS*-complemented Δ*kpsS*/Δ*waaF* double mutant (Δ*kpsS·waaF* + *kpsS* comp), and a *waaF*-complemented Δ*kpsS*/Δ*waaF* double mutant (Δ*kpsS·waaF* + *waaF* comp).

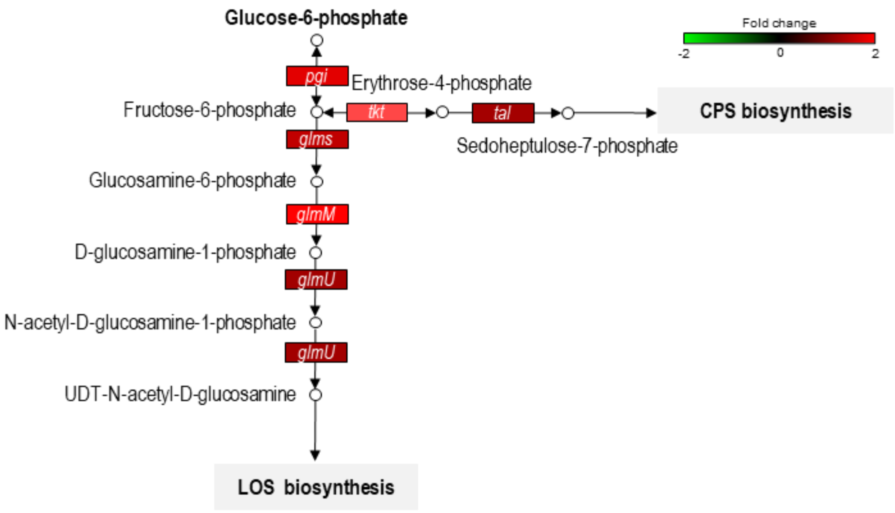

Fig. S5. Changes in the transcriptional levels of the genes involved in CPS and LOS synthesis under aerobic conditions. Transcriptional levels of genes involved in CPS and LOS biosynthesis in *C. jejuni* under aerobic conditions were assessed by RNA-Seq. Fold change was defined as $\mathrm{RPKM}_{\mathrm{aerobic\ conditions}}/\mathrm{RPKM}_{\mathrm{microaerobic\ conditions}}$. The diagram was prepared based on KEGG database (https://www.genome.jp/kegg-bin/show_pathway?cje01250).

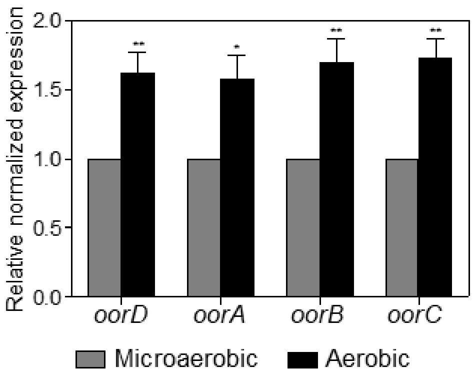

Fig. S6. Confirmation of the up-regulation of the *oor* genes under aerobic conditions using qRT-PCR. The transcriptional levels of *oorD*, *oorA*, *oorB* and *oorC* in the wild type were analyzed with qRT-PCR to confirm the results of RNA-Seq. The expression values were normalized using samples prepared under microaerobic conditions. The data present the means and the standard errors of the mean (SEM) of the results of three experiments. Statistical significance was determined with Student *t*-test compared to the results of microaerobic samples ($^{*}P < 0.05$, $^{**}P < 0.01$).

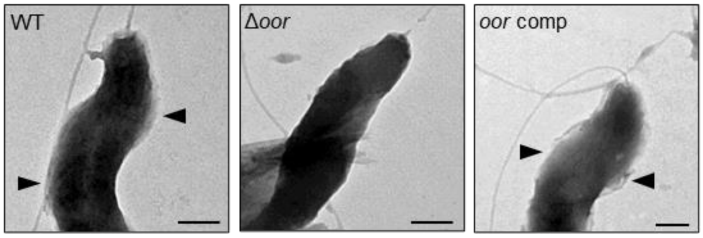

Fig. S7. Transmission electron microscopic (TEM) images of wild type (WT; *C. jejuni* NCTC 11168), an Δ*oor* mutant, and an *oor*-complemented strain after aerobiosis for 4 h. Black triangles indicate CPS and LOS. Scale bars represent 200 nm.

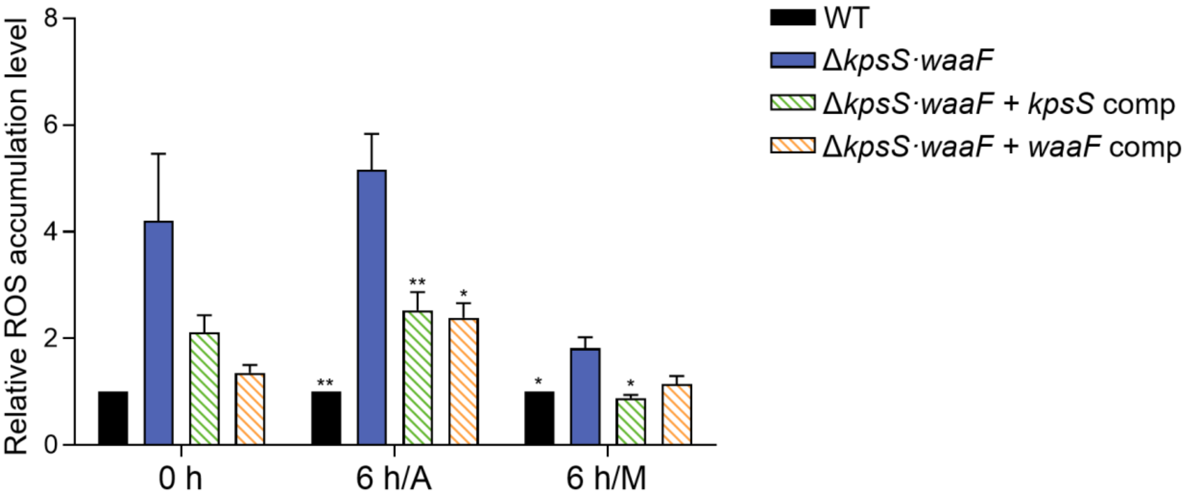

Fig. S8. ROS accumulation in the Δ*kpsS*/Δ*waaF* double mutant complemented with either *kpsS* or *waaF*. The relative levels of the total ROS accumulation in the *C. jejuni* strains before/after exposure to aerobic and microaerobic conditions for 6 h. A and M stand for aerobic and microaerobic conditions, respectively. The level of total ROS accumulation in WT was set as 1. A wild type (WT), a Δ*kpsS*/Δ*waaF* double mutant (Δ*kpsS·waaF*), a *kpsS*-complemented Δ*kpsS*/Δ*waaF* double mutant (Δ*kpsS·waaF* + *kpsS* comp), and a *waaF*-complemented Δ*kpsS*/Δ*waaF* double mutant (Δ*kpsS·waaF* + *waaF* comp). The data present the means and the standard errors of the mean (SEM) of the results of five experiments. Significance was assessed using one-way ANOVA ($^{*}P < 0.05$, $^{**}P < 0.01$).

Table S2. Primers used in this study

Primer	Sequence (5′-3′)	Reference
**Cloning**		
oor-SalI-F	AAAGTCGACCGCAGCAGAAGCAAAGACAA	This study
oor-BamHI-R	AAAGGATCCTCACTCTCATCTGCGATAGC	
oor-inverse-F	GGGTGTAAAATACGCCACTCAA	
oor-inverse-R	CTCATCTACCCAAACAGGGG	
Kan-F	GCGATGAAGTGCGTAAG	
Kan-R	CGGCTCCGTCGATACTATG	
oor-compl-F	AAATCTAGACACCGAGCATACGCCTGTAA	
oor-compl-R	ATTCTAGATTGGTTCAGATGCTAAAACATTACT	
kpsS-compl-F	TAGTCTAGACTTCCCTATCCATACAATAATTTCT	
kpsS-compl-R	TAGTCTAGACCGCTTTTCAAAATACAATGGCT	
waaF-compl-F	AAATCTAGAGCCCAAACCGACCAGCAAAAATGCC	
waaF-compl-R	AAATCTAGACTGATGGAGAAAAAGCTTGTTTTG	
**qRT-PCR**		
16s-RT-F	ATAAGCACCGGCTAACTCCG	(1)
16s-RT-R	TTCCATCTGCCTCTCCCTCA	
oorD-RT-F	TGTTAGCTATTGCCCCGCTG	This study
oorD-RT-R	GCCTCAGCAGTAAGTTTGGC	
oorA-RT-F	TCCATGACTGCAAGTAGCGG	
oorA-RT-R	ATCCCCATGAGTTGGTGCTT	
oorB-RT-F	ATGCCTACTCAATGGTGTTGG	
oorB-RT-R	GCCTGTTGCATAAGCTATTGCT	
oorC-RT-F	GCAGCCATTAAAGAAGGTCGT	
oorC-RT-R	CCTTCTTTTACACCCCCACG	

References

1. Kim J, Hur JI, Ryu S, Jeon B. 2021. Bacteriophage-mediated modulation of bacterial competition during selective enrichment of *Campylobacter*. Microbiol Spectr 9:e01703-21. https://doi.org/10.1128/spectrum.01703-21.
